# High prevalence of hepatitis B and HIV among women survivors of sexual violence in South Kivu province, eastern Democratic Republic of Congo

**DOI:** 10.1371/journal.pone.0292473

**Published:** 2024-07-03

**Authors:** Parvine Basimane Bisimwa, Giscard Wilfried Koyaweda, Dieudonné Bihehe Masemo, Rodrigue Balthazar Basengere Ayagirwe, Ahadi Bwihangane Birindwa, Patrick Ntagereka Bisimwa, Georges Kikuni Besulani, Théophile Mitima Kashosi, Cadeau Mugisho Matabishi, Bienfait Mitima Misuka, Jean Paulin Mbo Mukonkole, Jean Bisimwa Nachega, Denis Mukwege Mukengere, Narcisse Patrice Joseph Komas

**Affiliations:** 1 Viral Hepatitis Laboratory, Institut Pasteur de Bangui, Bangui, Central African Republic; 2 Faculty of Medecine, Université Evangélique en Afrique (UEA), Bukavu, Democratic Republic of Congo; 3 Panzi General Referral Hospital, Internal Medicine, Bukavu, Democratic Republic of Congo; 4 Molecular Biology Laboratory, Université Evangélique en Afrique (UEA), Bukavu, Democratic Republic of Congo; 5 International Center Advanced for Research and Training (ICART)/Panzi Fondation, Bukavu, Democratic Republic of Congo; 6 Departments of Epidemiology, Infectious Diseases and Microbiology, University of Pittsburgh School of Public Health, Pittsburgh, Pennsylvania, United States of America; 7 Departments of Epidemiology and International Health, Johns Hopkins Bloomberg School of Public Health, Baltimore, Maryland, United States of America; 8 Department of Medicine, Stellenbosch University Faculty of Medicine and Health Sciences, Cape Town, South Africa; Kwame Nkrumah University of Science and Technology, GHANA

## Abstract

**Introduction:**

Limited data are available on the prevalence rates of hepatitis B and acquired immunodeficiency syndrome (AIDS) among women survivors of sexual violence (WSSV) in South Kivu province, in the eastern part of the Democratic Republic of Congo (DRC), where armed conflicts persist. Here, we aimed to assess the prevalence of these two infections in this vulnerable local population.

**Methods:**

A total of 1002 WSSV, aged from 18 to 70 years old were enrolled from May 2018 to May 2020 at three healthcare facilities (Panzi, Mulamba and Bulenga hospitals), which are called “The One-Stop Centre Care Model" for the management of sexual violence in South Kivu. Blood samples were collected and tested for hepatitis B virus (HBV) and human immunodeficiency virus (HIV) antigens and antibodies using enzyme-linked immunoassay (ELISA) methods. Subsequently, viral load quantification for HBV and HIV were performed using the GeneXpert. Univariate and multivariate logistic regression models were used to assess factors associated with HIV-positive and HBV-positive status.

**Results:**

For HBV, overall prevalence was 8.9% (95% CI; 7.2–10.8%), 32.1% (95% CI; 29.3–35.0%), and 14.5% (95% CI; 12.3–16.8%) for HBsAg, anti-HBc and anti-HBs antibodies, respectively. Among the 89 HBsAg-positive patients, 17 (19.1%) were HBeAg-positive. The median age of individuals with a positive HBsAg test was higher than those with a negative test (median: 40 years (IQR 30–52) compared to 36 years (IQR 24–48)). Risk factors for HBV infection were age (≥35 years) (AOR = 1.83 [1.02–3.32]; *p* = 0.041), having no schooling (AOR = 4.14 [1.35–12.62]; *p* = 0.012) or only primary school-level (AOR = 4.88 [1.61–14.75]; *p* = 0.005), and multiple aggressors (AOR = 1.76 [1.09–2.84], *p* = 0.019). The prevalence of HIV was 4.3% [95% CI: 3.1–5.7%]. HIV/HBV co-infection occurred only in 5 individuals (0.5%). The HBV viral load was detectable (> 1 log_10_ UI/mL) in 61.8% of HBsAg-positive subjects and 64.8% HIV-positive subjects had a high viral load (≥ 3 log_10_ copies/mL).

**Conclusion:**

This study revealed a high prevalence of HBV and HIV infections among WSSV in South Kivu. The results generated highlight the urgent need for systematic screening of HBV and HIV by integrating fourth-generation ELISA tests in HIV and HBV control programs.

## Introduction

Sexually transmitted infections (STIs) constitute a heavy public health burden in many developing countries, including the Democratic Republic of Congo (DRC). Two STIs, i.e. those caused by hepatitis B virus (HBV) and human immunodeficiency virus (HIV), affect approximately 2 billion and 37.7 million people worldwide, respectively [1, 2]. HBV is one of the leading infectious killers globally, with 887,000 deaths due to HBV-associated hepatic sequelae such as acute hepatitis, liver cirrhosis, and liver cancer or hepatocellular carcinoma (HCC), recorded worldwide in 2015 [1]. Its prevalence is highest in the Western Pacific and African regions, where respectively 6.2% and 6.1% of the adult population is infected [3]. The World Health Organization (WHO) has classified the DRC as an intermediate endemicity area, with 5 to 7.99% of HBV infection [1, 3]. In endemic areas, HBV infection is usually transmitted from mother to child at delivery or by horizontal transmission among children, especially during the first five years [3–5]. It is also transmitted by sexual intercourse through vaginal and seminal secretions, by tattooing and piercing with non-sterilized equipment, by blood exposure accidents, and by other infected body fluids such as saliva [1, 3, 4].

The Joint United Nations Program on HIV/AIDS (UNAIDS) and WHO reports show that among the 37.7 million people living with HIV worldwide, adults (36.0 million) predominate. Of these infected adults, women, and girls (53%) were the most infected groups in 2020 [2, 5, 6]. According to the same reports, HIV infection was responsible for around 680,000 deaths worldwide in 2020, compared with 1.3 million in 2010 and 1.9 million in 2004, with a significant decrease (53%) in women and girls [2, 6]. However, despite the decrease in HIV incidence and associated mortality worldwide with increased access to potent antiretroviral therapy, incidence rates can nonetheless be alarming in post- or armed-conflict countries where sexual violence is used as weapon of war. In 2020, the WHO estimated that 30% of women have been raped, representing a vulnerable group that may be at high risk of HIV infection, particularly adolescent girls, and young women [6]. Sexual violence has become endemic in the eastern DRC, particularly in South Kivu [7]. According to the 2020 report of the Panzi Foundation, since 1999, the teams from the Panzi Hospital have taken care of over 55 000 women victims of sexual violence, among whom 12% were admitted within less than 72hours of the reported incident of sexual violence and 6% with pregnancies resulting from rape [8].

The South Kivu province, where this study was undertaken, is rife with frequent sexual violence perpetrated on women due to recurrent armed conflicts, especially in rural areas [7]. Furthermore, sporadic cases of sexual violence are reported in the city of Bukavu [9]. These situations may increase sexual transmission of HBV and HIV in this area due to their transmission mode. However, few studies have been conducted on the prevalence of HBV and HIV infections or the burden of HIV/HBV co-infection in South Kivu. This study was aimed to determine the prevalence and factors associated with HBV and HIV infections among WSSV in South Kivu. This information is important for predicting the risk of HBV/HIV co-infection and for setting up suitable management of the infections.

## Methods

### Study design, setting, and population

This was a cross-sectional study conducted from May 2018 to May 2020. The participants were recruited from three hospitals, two of which are located in rural areas (Bulenga and Mulamba Hospital), and one in the city of Bukavu (Panzi Hospital). These three hospitals, which are specialized in providing care for sexual violence, are called “The One Stop Centre Care Model" [9] and assist women who have been victims of sexual violence. This model allows victims and survivors of sexual violence to receive holistic care (medical, psychosocial, legal, and socio-economic reintegration) and other services to restore their health conditions [9]. Among these three hospitals, the Panzi Hospital is the reference center for the care of victims and survivors of sexual violence in South Kivu and in the DRC. This hospital receives 5 to 7 female victims of sexual violence every day through the Panzi Foundation and has partnered up with other one-stop centers, such as the Mulamba Hospital, located at about 70 km southwest of Bukavu in the rural area of Walungu, and the Bulenga Hospital, located near Goma, 165 km from Bukavu. The women were enrolled in this study through the above-mentioned hospitals when they came to receive various types of post-sexual violence care.

### Eligibility criteria

The study population included all women aged at least 18 years with a sexual violence record lasting more than 3 months and being monitored in one of the above-mentioned centers. They were admitted for management of various complications related to sexual violence including medical, surgical, gynecological, and social care. Women monitored for recent sexual violence (less than 3 months) or its complications were not included in this study. We screened 1230, but a total of 1002 women were enrolled in this study based on the sample size determined by using the HBV prevalence (5.0%) in the DRC [10], with a precision of ±3% at a 95% confidence level.

### Specimen and data collection

The enrollment of participants took place at the three healthcare facilities at different times during the study period. According to the policy for the management of victims of sexual violence in the region, cases reported in other clinics are transferred to the three one-stop centers for holistic care. At each One-Stop Centre, prior to administering a questionnaire, an information session was offered to WSSV providing clear explanations, in the local language where necessary, using readily understandable terms about HBV and HIV-related diseases. An informed-consent form was then signed by each participant and the collected information remained anonymous. Sociodemographic and clinical variables were recorded. Clinical variables mainly involved patient history of jaundice, vaccination, transfusion, scarification, tattoo, traditional excision, number of permanent and transient sexual partners before and after the sexual assault, number of aggressors, and the occurrence of pregnancy; sociodemographic variables included age, origin, tribe, level of schooling, religion, marital status, and occupation. Approximately 10 milliliters (mL) of venous blood was collected in an EDTA tube for rapid screening of HIV and HBV tests and results were reported to participants immediately after screening. The tubes containing the rest of the collected venous blood in the three “One-Stop Centre Care Model” were then centralized at the Medical Research Laboratory (MRL) at the Université Evangélique en Afrique (UEA) in Bukavu where the DBS were prepared by applying 4 spots of 75 microliters of each blood sample to appropriate Whatman papers strips. The spots were dried and sealed in plastic bags for storage at room temperature in the presence of a desiccant until their transfer to the Viral Hepatitis Laboratory at the Institut Pasteur de Bangui in the Central African Republic (CAR) for further analysis.

### Serological assays and viral load

The HBV markers were detected by enzyme-linked immunosorbent assay (ELISA) using DiaSource kits (DiaSource ImmunoAssays S.A., Belgium) according to the manufacturer’s instructions. All samples were tested for the hepatitis B surface antigen (HBsAg), and hepatitis B core antibody (anti-HBc) which represent the markers of infection by HBV and contact with HBV respectively. A confirmatory ELISA test of HBsAg was used for HBsAg-positive samples. HBsAg-negative samples were screened for the HBs antibody (anti-HBs Ab) to check the possibility of natural immunization, HBsAg-positive samples were screened for the hepatitis B e antigen/antibody (HBeAg/anti-HBe Ab) to check for the active replication of the virus. Anti-HBc IgM antibodies were tested as well for the HBsAg-positive samples. The Murex ELISA kit (DiaSorin S.p.A., Italy) was used for quality control and confirmation of all positive samples.

The presence of HIV was detected using the HIV Ab/Ag Combo ELISA kit (DiaSource ImmunoAssays S.A.) according to the manufacturer’s instructions. After serological testing, all HBV- and HIV-positive samples were transported to the Panzi Hospital laboratory for viral load quantification. This viral load for HBV DNA and HIV RNA were detected in the blood samples using Cepheid GeneXpert kits Xpert^®^ HBV Viral Load and Xpert^®^ HIV-1 Qual for HBV and HIV, respectively by following the manufacturer’s recommendations. The limit of detection for HIV RNA was 1.60 log_10_ which is 40 copies/mL and 1 log_10_ international units per milliliter (IU/mL) which is 10 IU/mL for HBV DNA.

### Statistical analysis

Data were collected from a structured survey questionnaire, compiled into a Microsoft Excel 2016 spreadsheet, and then imported into Stata SE 14.0 (Stata Corp LP, College Station, Texas, USA) for clean-up and analysis.

To describe the data, continuous variables were reported using the median along with the interquartile range (IQR) and categorical variables were summarized as frequencies and their percentages. To compare two means or medians, we used Student’s *t*-test. For HBV and HIV viral loads, medians were calculated only for detectable viral loads. We established three categories for hepatitis B viral loads: undetectable, low (< 3.30 log_10_ IU/mL which corresponds to < 2000 IU/mL), and high (≥ 3.30 log_10_ IU/mL which is ≥ 2000 IU/mL). For HIV, we defined two categories: low (< 3 log_10_ copies/mL which is <1000 copies/mL) and high (≥ 3 log_10_ copies/mL which is ≥ 1000 copies/mL). To compare proportions, we used the Pearson chi-square test or the Fisher exact test for proportions less than or equal to 5. We constructed a multivariable logistic regression model to assess factors associated with HIV-positive and HBV-positive status among WSSV and variables with a *p*-value ≤ 0.2 were included in the model.

The adjusted odds-ratios (AOR) and their 95% confidence intervals (CI) were derived to measure the strength of the association between the variables. All *p*-values were two-sided, and *p*-values < 0.05 indicated statistical significance.

### Ethical approval and consent to participate

This study obtained approval from the ethics committee of the Catholic University of Bukavu (UCB/CIE/NC/008/2016) reviewed in 2019 by the national health ethics committee (CNES001/DPSK/124PP/2019) and from the ethics and scientific committees of the Université de Bangui (22/UB/FACSS/CSVPR/19). Written informed consent was signed by the participant in the interest of knowing her serological status to consider early treatment in case of a positive result, as well as for her participation in the study. The preliminary results for HBV and HIV screening were given to participants immediately after screening and 3 weeks later for definitive results. HIV- and HBV-positive cases were referred to the Panzi Hospital for medical care.

## Results

### Sociodemographic and seroprevalence of HBV and HIV infection among survivors of sexual violence

**Fig 1:**
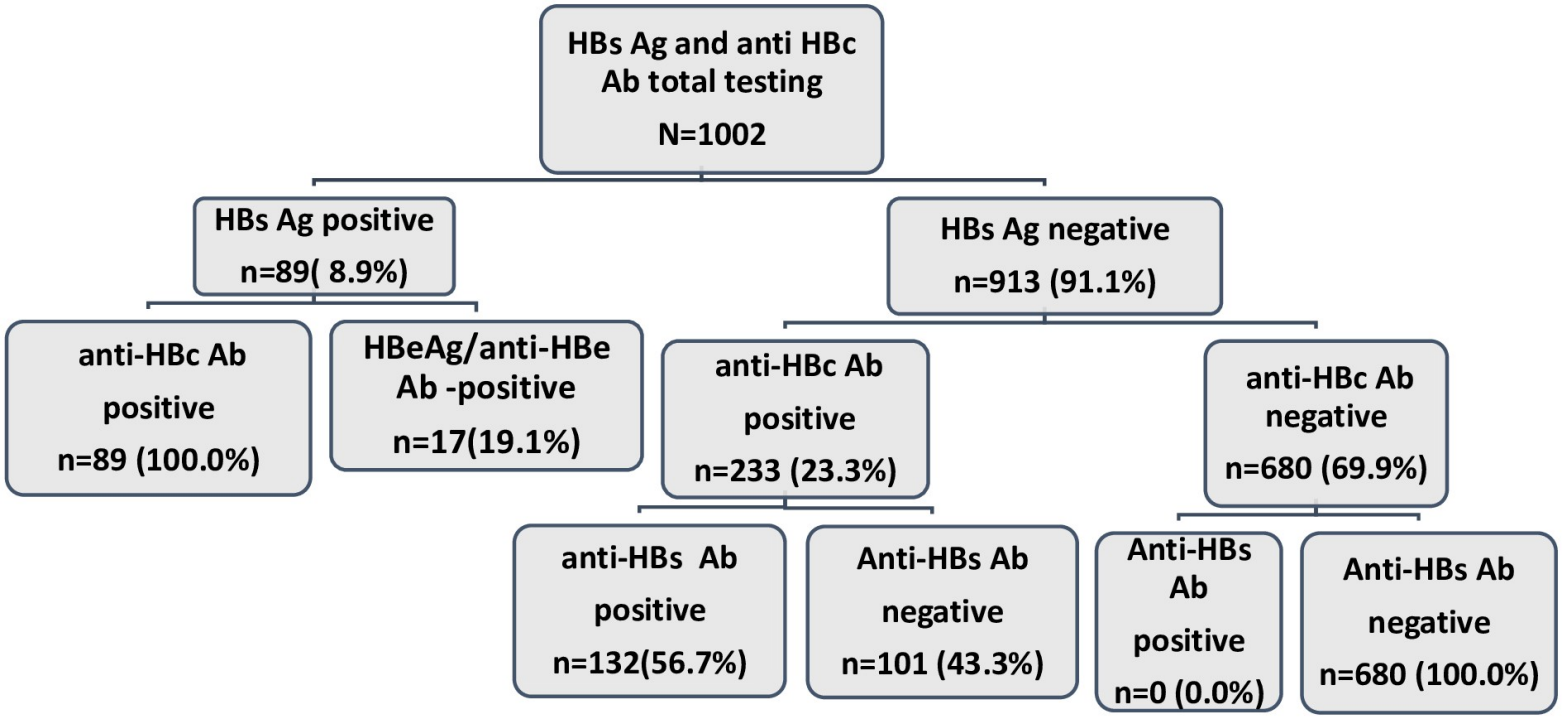
Testing of HBV markers.

Between May 2018 and May 2020, we screened 1 230 persons to enroll 1002 (81.5%) into our study. The median age of study participants was 37 years (IQ 25th and 75th, 25–48) and most were 35 years old and older (53.9%). Majority were married (47.7%), Protestant (53.5%) and from rural areas (92.4%) (Table 1). Of the 1002 participants, the prevalence rates of HBV markers were 8.9% (95% CI; [7.2–10.8%]), 32.1% [29.3–35.0%], and 14.5% [12.3–16.8%] for HBsAg, anti-HBc and anti-HBs antibodies, respectively. Of the 89 HBsAg-positive participants, 17 (19.1%) were also HBeAg/anti-HBe Ab -positive (Fig 1).

**Table 1:** Sociodemographic characteristics and prevalence of hepatitis B virus (HBV) and human immunodeficiency virus (HIV) among survivors of sexual violence in South Kivu.

Characteristics	Number	%	HBsAg			Anti-HBc antibodies			Anti-HBs antibodies			HIV Ag/Ab		
			Positive (n)	*%*	*p*	Positive (n)	%	*p*	Positive (n)	%	*p*	Positive (n)	%	*p*
**Overall prevalence**	1002	100	89 (1002)	8.9		322 (1002)	32.1		132 (913)	14.5		43 (1002)	4.3	
**Age (years)**														
<35	462	46.1	31 (462)	6.7	0.027	118 (462)	25.5	<0.001	48 (431)	11.1	0.007	20 (462)	4.3	0.957
≥35	540	53.9	58 (540)	10.7		204 (540)	37.8		84 (482)	17.4		23 (540)	4.4	
**Schooling level**														
No schooling	616	61.5	62 (616)	10.1	0.017	218 (616)	35.4	˂0.001	84 (554)	15.2	0.593	30 (616)	4.9	0.587
Primary	221	22.1	23 (221)	10.4		71 (221)	32.1		30 (198)	15.2		6 (221)	2.7	
Secondary	163	16.3	4 (163)	2.5		31 (163)	19.0		18 (159)	11.3		7 (163)	4.3	
University	2	0.2	0 (2)	0.0		2 (2)	100.0		0 (2)	0.0		0 (2)	0.0	
**Marital status**														
Single	235	23.5	17 (235)	7.2	0.064	56 (235)	23.8	0.015	24 (218)	11.0	0.353	7 (235)	3.0	0.507
Married	519	518	44 (519)	8.5		183 (519)	35.3		75 (475)	15.8		27 (519)	5.2	
Separated/Divorced	76	7.6	13 (76)	17.1		28 (76)	36.8		11 (63)	17.5		3 (76)	3.9	
Widowed	172	17.2	15 (172)	8.7		55 (172)	32.0		22 (157)	14.0		6 (172)	3.5	
**Occupation**														
No occupation	174	17.4	13 (174)	7.5	0.922	55 (174)	31.6	0.706	29 (161)	18.0	0.323	6 (174)	3.4	0.537
Farmer	667	66.6	60 (667)	9.0		222 (667)	33.3		88 (607)	14.5		32 (667)	4.8	
School /Pupil/ Student	105	10.5	11 (105)	10.5		28 (105)	26.7		9 (94)	9.6		3 (105)	2.9	
Entrepreneur (trade)	48	4.8	4 (48)	8.3		14 (48)	29.2		6 (44)	13.6		1 (48)	2.1	
Public sector worker	8	0.8	1 (8)	12.5		3 (8)	37.5		0 (7)	0.0		1 (8)	12.5	
**Tribe**														
Shi	478	47.7	28 (478)	5.8	<0.001	127 (478)	26.6	0.009	61 (450)	13.4	0.680	19 (478)	4.0	0.018
Havu	184	18.4	35 (184)	19.0		82 (184)	44.6		23 (149)	15.4		5 (184)	2.7	
Fuliru	82	8.2	7 (82)	8.5		28 (82)	34.1		9 (75)	12.0		10 (82)	12.2	
Lega	74	7.4	7 (74)	9.4		24 (74)	32.4		7 (67)	10.4		4 (74)	5.4	
Bembe	45	4.5	2 (45)	4.4		18 (45)	40.0		10 (43)	23.3		1 (45)	2.2	
Tembo	54	5.4	5 (54)	9.3		16 (54)	29.6		7 (49)	14.3		1 (54)	1.9	
Other	85	8.5	5 (85)	5.9		27 (85)	31.8		15 (80)	18.7		3 (85)	3.5	
**Religion**														
No religion	7	0.7	1 (7)	14.3	0.071	4 (7)	57.1	0.379	1 (6)	16.7	0.703	0 (7)	0.0	0.632
Catholic	429	42.8	40 (429)	9.4		139 (429)	32.4		57 (389)	14.7		22 (429)	5.1	
Muslim	5	0.5	2 (5)	40.0		3 (5)	60.0		0 (3)	0.0		0 (5)	0.0	
Protestant	536	53.5	42 (536)	7.8		167 (536)	31.2		73 (494)	14.8		21 (536)	3.9	
Other	25	2.5	4 (25)	16.0		9 (25)	36.0		1 (21)	4.8		0 (25)	0.0	
**Geographic area**														
Rural	926	92.4	79 (926)	8.5	0.173	296 (926)	32.0	0.687	120 (847)	14.2	0.365	41 (926)	4.4	0.458
Urban	76	7.6	10 (76)	13.2		26 (76)	34.2		12 (66)	18.2		2 (76)	2.6	

Ab, antibodies; Ag, antigens; HBs Ag, hepatitis B surface antigens; HBc, hepatitis B core

The median age of individuals with a positive HBsAg test was higher than those with a negative test (median: 40 years (IQR 30–52) compared to 36 years (IQR 24–48). Furthermore, participants aged 35 years and older were more frequently HBsAg-positive than those under 35 years (*p* = 0.027). A statistical difference was observed in participants with no formal schooling or only primary-school education (10.1% and 10.4%) compared with those with secondary- or university-level education (2.5% and 0.0%); p = 0.017. However, there was no statistical difference in the prevalence of HBV infection according to marital status or occupation, despite the slightly higher number of infection cases among separated/divorced women and public sector workers (17.1 and 12.5%; *p* = 0.064 and *p* = 0.922, respectively). Participants from the Havu tribe showed higher prevalence compared with participants from other tribes (19.1%; *p*<0.001). Religion and geographic origin of participants were not statistically associated with HBsAg positivity.

Similarly, for anti-HBc antibodies, the over-35 age group had a high prevalence compared with the under-35 age group (37.8% vs. 25.5%; *p*<0.001). For anti-HBs antibodies, a statistical difference was observed in the over-35 age group compared with those under 35 years of age (17.4% vs. 11.1%; *p* = 0.007).

A total of 43 of the 1002 participants (4.3% [3.1%–5.7%]) tested positive for HIV. The Fuliru tribe was more frequently associated with HIV infection than the other tribes (12.2%; *p* = 0.018). Although HIV infection appeared high, it was not statistically significant among women aged 35 years and over (4.4%), with no schooling (4.9%), married (5.2%), public sector workers (12.5%), Catholic (5.1%), or from rural areas (4.4%) (Table 1). Regarding HBV-HIV co-infection, the prevalence was 0.5%.

### Clinical history, and HBV and HIV infections among survivors of sexual violence

For HBV infection, a statistically significant association was found between the presence of HBsAg and multiple aggressors (11.0%; *p* = 0.022). Multiple aggressors were represented by the act of sexual assault by two or more aggressors, whether in a single assault or in multiple sexual assaults. The absence of HBV vaccination was statistically associated with the presence of HBsAg (*p* = 0.011). There was no statistical difference between the prevalence of HBV infection and other factors. However, participants with a history of tattoo, traditional genital excision, multiple sexual partners before sexual violence and current multiple sexual partners had slightly higher prevalences of HBV (10.3%, 10.6%, 10.0%, and 9.1%, respectively) (Table 2).

**Table 2:** Clinical history and hepatitis B virus (HBV) and human immunodeficiency virus (HIV) infections among women survivors of sexual violence.

Characteristics	Number	HBsAg			HIV Ag/Ab		
	N (%)	Positive n (%)	Negative n (%)	*p-value*	Positive n (%)	Negative n (%)	*p-value*
**Pregnancy**							
Yes	61 (6.1)	5 (8.2)	56 (91.8)	0.846	2 (3.3)	59 (96.7)	0.687
No	941 (93.9)	84 (8.9)	857 (91.1)		41 (4.4)	900 (95.6)	
**History of jaundice**							
Yes	23 (2.3)	2 (8.7)	21 (91.3)	0.975	1(4.3)	22 (95.7)	0.989
No	979 (97.7)	87 (8.9)	892 (91.1)		42 (4.3)	937 (95.7)	
**Vaccination**							
Yes	6 (0.6)	0 (0.0)	6 (100.0)	0.011	0(0.0)	6 (100.0)	0.603
No	996 (99.4)	89 (8.9)	907 (91.1)		43 (4.3)	953 (95.7)	
**Transfusion**							
Yes	47 (4.7)	1 (2.1)	46 (97.9)	0.095	1 (2.1)	46 (97.9)	0.453
No	955 (95.3)	88 (9.2)	867 (90.8)		42 (4.4)	913 (95.6)	
**Condom use**							
Never	974 (97.2)	88 (9.0)	886 (91.0)	0.316	41 (4.2)	933 (95.8)	0.450
Yes	28 (2.0)	1 (3.5)	27 (96.5)		2(7.1)	26(92.9)	
**Tattoo**							
Yes	39 (3.9)	4 (10.3)	35 (89.7)	0.758	2 (5.1)	37 (94.9)	0.793
No	963 (96.1)	85 (8.8)	878 (91.2)		41 (4.3)	922 (95.7)	
**Scarification**							
Yes	28 (2.8)	2 (7.1)	26 (92.9)	0.743	0 (0.0)	28 (100.0)	0.256
No	974 (97.2)	87 (8.9)	887 (91.1)		43 (4.4)	931 (95.6)	
**Traditional excision**							
Yes	123 (12.3)	13 (10.6)	110 (89.4)	0.483	4 (3.3)	119 (96.7)	0.544
No	879 (87.7)	76 (8.6)	803 (91.4)		39 (4.4)	840 (95.6)	
**Multiple sex partners before sexual violence**							
Yes	609 (60.8)	61 (10.0)	548 (90.0)	0.174	28 (4.6)	581 (95.4)	0.633
No	393 (39.2)	28 (7.1)	365 (92.9)		15 (3.8)	378 (96.2)	
**Current multiple sex partners**							
Yes	536 (53.5)	49 (9.1)	487 (90.9)	0.824	20 (3.7)	516 (96.3)	0.354
No	466 (46.5)	40 (8.6)	426 (91.4)		23 (4.9)	443 (95.1)	
**Number of aggressors**							
Single	521 (52.0)	36 (6.9)	428 (93.1)	0.022	21 (4.0)	499 (96.0)	0.672
Multiple	481 (48.0)	53 (11.0)	428 (89.0)		22 (4.6)	459 (95.4)	

Ab, antibodies; Ag, antigens; HBs Ag, hepatitis B surface antigens; HBc, hepatitis B core; virus; HIV, human immunodeficiency virus.

For HIV infection, no association was found with those factors.

### Factors associated with HBV infection

After controlling for other potential correlates, we found that age 35 years or older (AOR = 1.83; 95% CI: 1.02–3.32), having no schooling (AOR = 4.14; 95% CI: 1.35–12.62) or primary schooling only (AOR = 4.88; 95% CI: 1.61–14.75), being part of the Havu tribe (AOR = 4. 84; 95% CI: 1.08–21.64), and multiple aggressors (AOR = 1.76; 95% CI: 1.09–2.84) remained associated with prevalent HBV infection (Table 3). For HIV infection, no associated factors were found.

**Table 3:** Multivariable analysis of factors associated with HBs Ag positive among survivors of sexual violence.

Characteristics	**AOR [95% CI]	*p*-value
**Age group (years)**		
≥35	1.83 [1.02–3.32]	0.041
**Schooling level**		
None	4.14 [1.35–12.62]	0.012
Primary	4.88 [1.61–14.75]	0.005
**Aggressors**		
Multiple	1.76 [1.09–2.84]	0.019
**Tribe**		
Havu	4.84 [1.08–21.64]	0.039

**AOR: Adjusted odds ratio.

### Level of HBV and HIV viral loads in positive participants

Out of a total of 89 HBsAg-positive samples for hepatitis B virus, the median viral load for detectable samples (n = 55) was 2.81 log_10_ IU/mL (IQR: 2.28–5.08); 38.2% of the samples had an undetectable viral load, 39.3% a low viral load (˂ 3.30 log_10_ IU/mL), and 22.5% a high viral load (≥ 3.30 log_10_ IU/mL). For HIV, for those participants who tested positive for the first time, the median viral load was 4.26 log_10_ copies/mL (18 000 copies/mL) (IQR 2.47–5.12) (Table 4). The distribution of the HIV viral load showed a low (˂ 3 log_10_ copies/mL) viral load for 35.2% of the patients and a high with (≥ 3 log_10_ copies/mL) for 64.8% (Table 4).

**Table 4:** HBV and HIV viral load results in participants who tested positive.

Virological parameters	Number	%
**HBV DNA viral load (IU/mL)**	**N = 89**	
Median **(IQR25-IQR75) for detectable (log**_**10**_ **IU/mL)**	2.81 (2.28–5.08)	
Undetectable	34	38.2
Low	35	39.3
High	20	22.5
**HIV RNA viral load (copies/mL)**	**N = 34**	
Median **(IQR25-IQR75) log**_**10**_ **copies/mL**	4.26 (2.47–5.12)	
Low	12	35.2
High	22	64.8

## Discussion

In this study, the overall prevalence of HBsAg was 8.9% [7.2%-10.8%] and 32.1% [29.3%–35.0%] for anti-HBc antibody. These results demonstrate the high burden of HBV in this vulnerable population. The prevalence of HBV infection varies across regions and different types of population in the DRC [10]. Previous studies on HBV infection have focused on blood donors in DRC different regions, reporting HBsAg prevalence rates ranging from 6.8% to 9.2%, but in other population groups, prevalence seems to be much lower [11–17]. For example, one study in the Maniema province reported a prevalence of 5.9% for HBsAg in pregnant women [18]. However, the results of the 2013–2014 Demographic and Health Survey on the general population showed a prevalence of 3.3% in adults and 2.2% in children [10]. In contrast, an HBsAg prevalence of 24.6% was reported on samples from 2003 to 2012 in the yellow fever surveillance program from patients with acute febrile jaundice and suspected yellow fever [19].

The difference in hepatitis B prevalence rates in these different regions of the DRC can likely be attributed to the exposure to different risk factors according to population type, and probably arises from the absence of a viral hepatitis control and prevention program, as well as the fact that hepatitis B vaccination is limited to infants only. This variation in prevalence is also found in sub-Saharan Africa, where the rate of HBV infection, which is often very high, also varies with the country, the studied populations, and the used techniques [20–37]. For example, the overall prevalence of 42.3% for anti-HBc antibody with 15.5% for HBsAg in students with high sexual activity in Bangui [23].

Our investigation also revealed that 14.5% of the study population showed anti-HBs antibodies. Of the participants with this immunization marker, only 11.4% had a history of hepatitis B vaccination when tested within 72 h of sexual violence, and 88.6% had no history of previous hepatitis B vaccination. Later, the total anti-HBc antibody was present, clearly demonstrating that they had been in contact with the virus itself. An association was found between the rate of immunization against HBV infection and the over-35 years age group. Therefore, it is difficult to demonstrate precisely when the infection was acquired with respect to sexual abuse but given the high proportion of antibody detection in older patients, the hypothesis of infection acquired in conjunction with sexual abuse is highly plausible. Unfortunately, the antibody titer was not determined in this study. The anti-HBs antibody titer for HBV has been shown to decrease with age according to the age of the infection. Thus, the proportion of older individuals with anti-HBs antibodies at ≥10 IU/mL may be low if the infection was acquired in childhood, although they may nevertheless remain immunized against the infection [38].

For HIV infection, a prevalence of 4.3% [3.1%–5.7%] was found using fourth-generation ELISA tests. This prevalence is very high for this population category compared to that reported in a previous study among blood donors in Bukavu, which showed a low prevalence using rapid tests [12]. However, before the introduction of blood safety protocols in Kisangani, a prevalence of 4.7% was reported in this same group [15]. Nevertheless, our study demonstrates, the high prevalence of HIV among sexually abused patients in South Kivu province.

The link between sexual violence against women and girls and HIV acquisition has been shown in several studies [39]. For example, in Uganda, 15–49-year-old women who had experienced sexual violence showed a 1.6 fold increase in risk of HIV infection compared with those who had never experienced violence [40]. Similarly, in a study in South Africa, young women aged 15–24 years who reported multiple episodes of sexual violence had a 1.5-fold increase in risk of acquiring HIV than women with one or no episodes of sexual violence; furthermore, an estimated 12% of new HIV infections could be attributed to sexual violence [41].

The same findings were reported in a cross-sectional study of 28,000 married women aged 15–49 years in India, with a 4-fold higher risk of being HIV-positive among those who had experienced physical and sexual violence by their partners than those who had not [42]. Although studies of HIV among women who have experienced sexual violence are rare in the DRC, but HIV prevalence is often very high (7.5%) among female sex workers. This high prevalence contrasts with the overall HIV incidence in the general population, which is estimated to have decreased from 2010 to 2020 from 1.2% to 0.7%, with a national prevalence of 1.1% among women aged 15–49 years [43]. Furthermore, the prevalence may be underestimated considering the low rate of HIV testing in different groups and key populations, the use of rapid tests in routine screening, and diagnoses that sometimes produce false negatives with a risk of undetected HIV cases [44]. It has been well documented that women are two to four times more likely than men to contract the virus through unprotected vaginal penetration [45–47]. Sexual abuse of women and girls by one or more individuals is a direct risk factor for HIV transmission. The risk factors associated with HIV infection observed in this study were as follows: age less than or equal to 25 years, belonging to the Fulero tribe, occasional condom use, and number of assailants greater than or equal to 5. Women with a history of sexual assaults with more than 5 men in their lifetime had a higher frequency of HIV infection than those with fewer than 5 abusers. These results are consistent with studies that have shown that HIV is more prevalent among young women, sex workers, those with multiple sexual partners, those who have experienced physical or sexual violence, and those with low levels of education, but other factors such as poverty and early marriage are also implicated [47–50].

The main sources of HIV transmission vary by country. Risks found in sexual behavior include age at first intercourse [51], being sexually active, having multiple sexual partners, having unprotected sex, having unprotected sex with strangers [52], having sex during or after alcohol use [53], sexual violence [45], and transactional sex [54].

Addressing violence against women and gender equality has been identified by UNAIDS in the AIDS Investment Framework as essential for an effective response to the HIV epidemic [55]. Addressing gender-based violence (GBV) could have a significant impact on the HIV epidemic. Indeed, the national HIV program needs to recognize the following aspects: the direct and indirect implications of GBV in the mechanism of HIV dissemination, the importance of perpetrator dynamics, and that GBV reduction should be part of HIV prevention programs. Effective interventions are likely to include a structural component and a sexual violence awareness component.

Our study also revealed the prevalence of HBV/HIV co-infection which was 0.5%. This prevalence appears to be low, because most studies on co-infection in sub-Saharan Africa always report high prevalence rates [56]. Nevertheless, this prevalence varies across regions defined by HBV endemicity. In regions such as sub-Saharan Africa and East Asia, where HBV prevalence is high, most HBV infections occur perinatally or in early childhood through close household contact, medical procedures, or cultural practices such as scarification or tattooing [57]. HBV infections are therefore more likely to progress to chronic infections, resulting in a high prevalence of chronic HBV infection among young people at risk for sexual HIV [58].

HBV and HIV quantifications were performed on samples from our study populations using the automated GeneXpert^®^ system. The HBV viral load results showed that 77.5% had undetectable or low viral load and 22.5% had a high viral load (≥ 3.30 log_10_ IU/mL). The 77.5% for whom the viral load was undetectable or low were not likely to be put on antiviral therapy. However, the 22.5% with high viral load should be treated therapeutically for infection, following further testing. This quantification test must be made available and accessible to all patients in South Kivu to avoid shortages of antivirals or high expenses related to the routine therapeutic management of anyone infected with HBV. Indeed, it is known that more than 90% of infected adults spontaneously recover from acute viral hepatitis B, with the disappearance of HBsAg after about 6 months [59, 60]. Accurate measurement of HBV DNA levels in the blood is essential to diagnose HBV infection, establish the prognosis of HBV-related liver disease, and guide therapeutic decision-making to determine whether to initiate antiviral therapy and monitor the virological response to antiviral therapy and the emergence of resistance [61, 62].

In resource-limited countries such as the DRC, the ability to determine the HBV viral load is a major issue for the therapeutic management of patients, because the technical facilities recommended by international guidelines are not available and/or accessible [63]. Quantifying HBV DNA in the blood is one of the essential tests to establish the prognosis of infection and support any therapeutic decisions [61, 62].

Of the 34 subjects in whom the HIV viral load was determined, 64.8% had a high viral load (≥ 3 log_10_ copies/mL) and 35.2% had a low viral load (< 3 log_10_ copies/mL). These viral loads show that HIV testing is still a real problem in this area due to the inaccessibility of ELISA tests, which clearly have added value for the detection and diagnosis of HIV cases. The dearth of modern technical facilities contributes to the underestimation of HIV cases following the use of rapid tests. In this study, the positive cases had a detectable HIV viral load, except for nine cases that were not tested due to the insufficient amount of blood sample.

Our results clearly show the importance of using comprehensive tests for the diagnosis of HBV and HIV in a vulnerable population group. However, not all tests to establish a complete diagnosis of liver diseases, such as transaminase and other complementary tests, were carried out, which thus constitutes a limitation of this study. Determining viral load remains an essential tool for the management of HBV infection.

## Limitations

Given that our study is cross-sectional, we cannot definitively demonstrate that these correlations are causal, as we cannot necessarily determine which occurred first.

In this study, we did not include women admitted to the three centers who reported sexual violence occurring within the past 72 hours, because once they arrive at one of the centers within 72 hours of the assault, they are provided with prevention measures against HBV and HIV. However, beyond 72 hours, no prophylaxis is given.

## Conclusion

This study shows a high prevalence of HBV and HIV infections among girls and women who have survived sexual violence in South Kivu, despite the low national prevalence of HIV and the intermediate endemicity of HBV infection in the DRC. In this study, the ELISA technique revealed that the prevalence of these infections is underestimated in South Kivu where girls and women have been exposed to sexual violence for many years due to repetitive wars. We suggest that actions to combat sexual violence against girls and women be integrated into strategies to combat HIV/AIDS and viral hepatitis B.

## Supporting information

S1 File (XLSM)
